# Case Report: A rare case of ALK-KIF5B gene fusion benefited from treatment with lorlatinib

**DOI:** 10.3389/fonc.2025.1594072

**Published:** 2025-08-07

**Authors:** Yuxi Luo, Weiwei Ouyang

**Affiliations:** ^1^ Department of Oncology, Affiliated Hospital of Guizhou Medical University, Guiyang, China; ^2^ Teaching and Research Section of Oncology, Guizhou Medical University, Guiyang, China; ^3^ Department of Oncology, Affiliated of Cancer Hospital of Guizhou Medical University, Guiyang, China

**Keywords:** KIF5B-ALK gene fusion, non-small cell lung cancer, anaplastic lymphoma kinase inhibitors, loratinib, case report

## Abstract

The anaplastic lymphoma kinase (*ALK*) gene encodes a transmembrane receptor tyrosine kinase. Most mutations in *ALK* gene result from translocations with other genes, forming fusion oncogenes. To date, 21 different genes have been identified as *ALK* fusion partners, each activating distinct signaling pathways that influence cancer cell proliferation, invasiveness, and tumorigenicity. *ALK* tyrosine kinase inhibitors (ALK-TKIs) have demonstrated significant efficacy in *ALK*-positive non-small cell lung cancer (NSCLC) and are widely utilized as first-line therapy. Lorlatinib, a third-generation ALK inhibitor, is effective in both treatment-naïve and previously treated patients with advanced NSCLC, exhibiting strong systemic and intracranial antitumor activity. This report presented a case of lung adenocarcinoma with 51 genetic variants, including a rare fusion variant: exon 15 of *KIF5B* fused to exon 20 of *ALK*, KIF5B-ALK (K15:A20). Following lorlatinib treatment, partial remission was achieved, and disease stability was maintained for an extended period, suggesting a favorable response to therapy. This case highlighted the potential sensitivity of the *KIF5B-ALK* (K15:A20) fusion to lorlatinib and the need for further investigation into lorlatinib’s efficacy across different *KIF5B-ALK* fusion variants. Additionally, other fusion types and treatment options for *KIF5B-ALK* fusions with varying breakpoints were discussed.

## 1 Introduction

Non-small cell lung cancer (NSCLC) accounts for approximately 80-85% of all lung cancer cases, and lung cancer-related mortality represents 18.7% of all cancer deaths worldwide in 2022 (1). The overall 5-year survival rate for lung cancer is 19.7%. Among stage I NSCLC patients, the 5-year survival rate ranges from 77% to 92% after surgery, whereas for stage III–IV NSCLC patients, it drops to 17–36% (2). In the Chinese population, *ALK* fusion mutations occur in approximately 5.5% of NSCLC cases (3). Among them, the echinoderm microtubule-associated protein-like 4 (*EML4-ALK*) fusion is the most common, comprising approximately 85% of *ALK* fusion mutations. *ALK* fusions are the second most prevalent driver mutations in NSCLC after *EGFR*-sensitive mutations, and *EML4-ALK* served as a key therapeutic target. ALK-TKIs have demonstrated significant efficacy in treating NSCLC patients with *ALK* rearrangements. *ALK* gene fusions are primarily found in adenocarcinoma subtypes of non-small cell lung cancer (NSCLC), mainly co-occurring with mutations in other genes, such as *TP53* and *BIM*. ALK alterations are more frequently identified in younger, non-smoking patients with advanced lung adenocarcinoma, and are associated with a higher propensity for brain metastases (4, 5). In addition to *EML4*, other *ALK* fusion partners include *TRK, KIF5B, KLC1, TFG, TPR, HIP1, STRN, DCTN1, SQSTM1, NPM1, BCL11A*, and *BIRC6* (6). *KIF5B* is one such fusion partner, with multiple possible fusion breakpoints. In this case, exon 15 of *KIF5B* is fused to exon 20 of *ALK*. The *KIF5B-ALK* fusion was first described in 2009 by Takeuchi et al. (7), who reported a fusion between Intron 24 of *KIF5B* and intron 19 of *ALK*. Wong et al. (8) later identified the *KIF5B-ALK* (K15:A20) fusion mutation, corresponding to the variant observed in this case. NSCLC patients with *ALK* fusion have exhibited significant responses to ALK-TKIs. In phase I trials, crizotinib, the first-generation ALK-TKI, achieved an objective response rate (ORR) of 57% in *ALK*-positive advanced NSCLC, leading to its approval as the first ALK inhibitor for clinical use (9). Since 2014, second-generation ALK-TKIs have emerged, exhibiting improved efficacy. Compared with crizotinib, the second-generation ALK-TKIs alectinib and brigatinib have demonstrated longer median progression-free survival (PFS) of 34.8 and 30.8 months, respectively. Among ALK-TKIs, the third-generation inhibitor lorlatinib has shown the longest median PFS (10–12). A Canadian cohort study showed that the median OS of patients receiving multi-line treatment including the third-generation TKI was significantly longer than that of patients receiving only monotherapy (55 months vs. 26 months, HR=4.64, p < 0.0001) (13). According to the 2024 NCCN guidelines, alectinib, brigatinib, or lorlatinib are recommended as first-line monotherapies for patients with *ALK*-positive metastatic NSCLC. The case presented in this article highlights the importance of developing individualized treatment plans for patients with rare gene mutations.

## 2 Case presentation

A 53-year-old Chinese female, with no history of smoking or family history of related tumors, was admitted to the Cancer Hospital of the Chinese Academy of Medical Sciences with symptoms of hemiplegia, headache, and mental distress. On January 28, 2023, magnetic resonance imaging (MRI) revealed multiple abnormal signal foci in the brain parenchyma, the largest measuring approximately 24 × 21 mm², with a rightward midline shift and compression of the left lateral ventricle. Concurrently, chest-abdomen computed tomography (CT) identified a 38 × 33 mm² subpleural mass in the anterior segment of the upper lobe of the right lung. Multiple nodules of varying sizes were found in both lungs and bilateral pleura, the largest measuring approximately 10 × 8 mm². Additionally, two hepatic lesions were detected, measuring approximately 20 × 15 mm² and 44 × 55 mm², respectively. Based on the 9th Edition of the TNM staging system for lung cancer established by the International Association for the Study of Lung Cancer (IASLC), the patient was staged as cT3N2bM1c2 IVB. To relieve symptoms and improve quality of life, the neurosurgery department recommended surgical intervention. After completing the necessary preoperative evaluations and excluding contraindications, the patient underwent “resection of the left central frontal lobe occupying lesion with bone flap reduction and fixation” on February 1, 2023. Frozen-section analysis of the left frontal lobe mass indicated a poorly differentiated malignant tumor with large cell atypia and evident mitotic activity. Postoperative pathology confirmed poorly differentiated carcinoma infiltrating brain tissue, exhibiting solid, sheet-like growth patterns. Immunohistochemistry and imaging findings were consistent with brain metastasis from primary lung adenocarcinoma.

**Figure 1:**
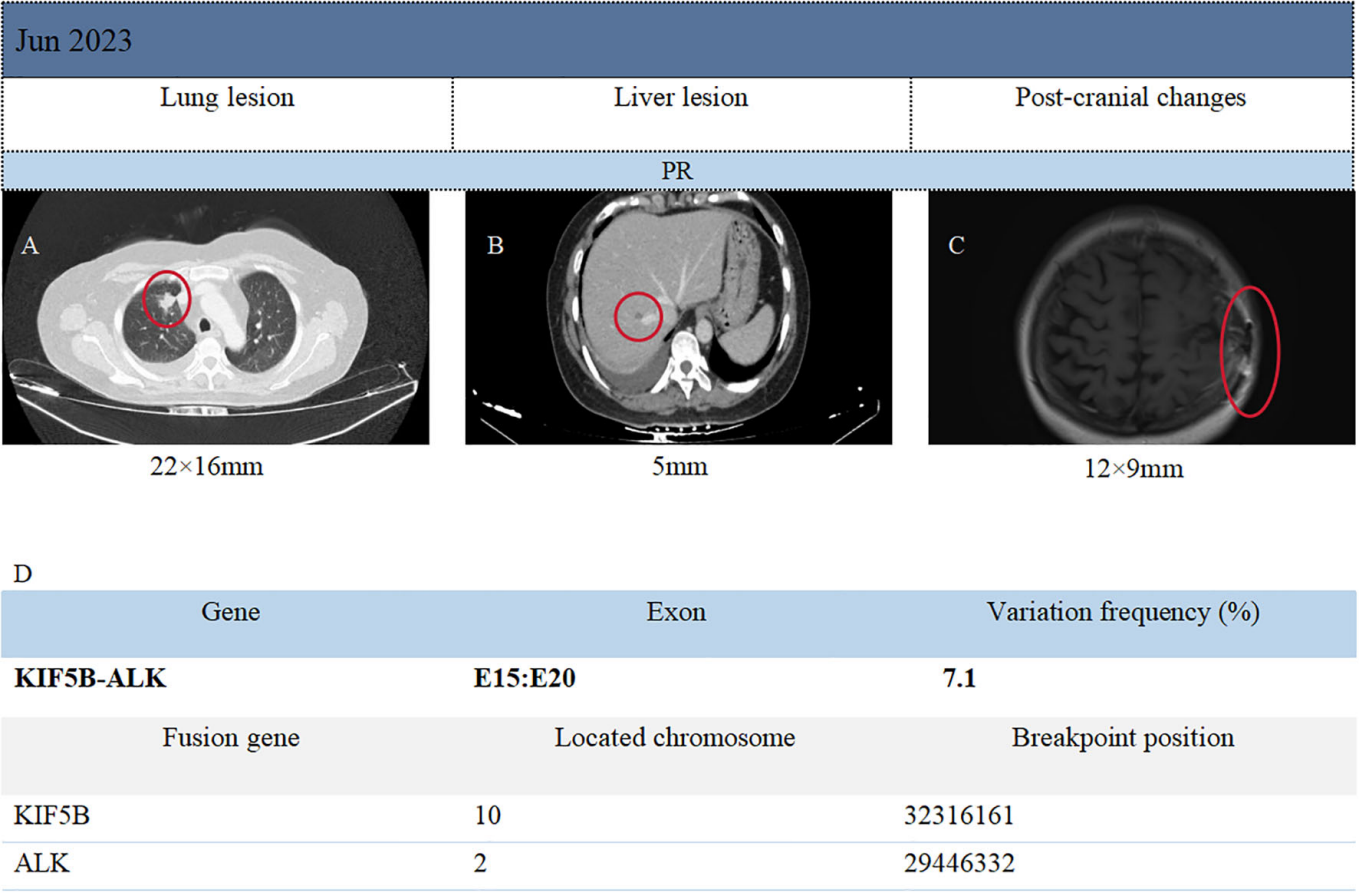
Imaging surveillance of primary and metastatic lesions, as well as presentation of genetic test results.

**Figure 2:**
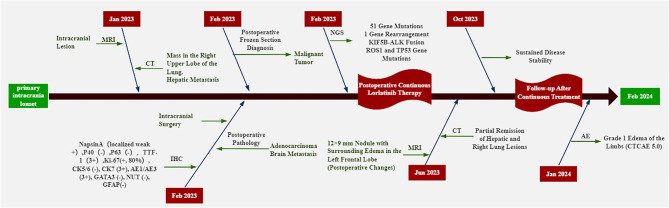
The timeline of the treatment.

Immunohistochemical results included: Napsin A (localized weak +), P40 (-), TTF-1 (3+), Ki-67 (80%+), CK5/6 (-), CK7 (3+), AE1/AE3 (3+), GATA3 (-), NUT (-), and GFAP (-). The PD-L1 value of the patient was not detected throughout the treatment. Capture-based next-generation sequencing (NGS) identified 51 gene mutations and one gene rearrangement, including a *KIF5B-ALK* (K15:A20) fusion, as well as *TP53* (c.255del) and *ROS1* (c.5912G). The ROS1 c.5912G>C mutation detected in this patient is currently classified as a variant of unknown significance (type 4 mutation). At present, the clinical relevance of this mutation is considered limited. Therefore, the ROS1 mutation detected in this case is not deemed to have clear clinical significance. The genetic testing was conducted by Genetron Health (**Figure 1D**). Based on the above examination results, the patient was discharged from the hospital after undergoing head surgery in February 2023 and received oral loratinib (100 mg daily) targeted therapy until June 2023. On June 5, 2023, the patient visited Guizhou Cancer Hospital for a follow-up examination. The CT scan revealed a 22 × 16 mm^2^ nodule in the upper tip segment of the right lung (**Figure 1A**), multiple nodules in the left lung, and a 5 mm nodule in segment S8 of the liver (**Figure 1B**). MRI of the head displayed a 12 × 9 mm^2^ nodule in the left frontal lobe with surrounding edema, aligning with postoperative changes (**Figure 1C**). Continued treatment up to October 16, 2023 demonstrated stable disease (SD) in the brain, liver, and upper lobe of the right lung compared to June. In June 2023, four months post-surgery and lorlatinib treatment, partial remission (PR) of intrahepatic, intracranial, and right lung lesions was achieved. Disease stability was maintained at eight months of follow-up with lorlatinib therapy (RECIST 1.1), with grade 1 limb edema (CTCAE 5.0) as the only reported adverse event (AE). Patient clinical management workflow is shown in **Figure 2**.

## 3 Discussion

### 3.1 *KIF5B* gene fusion and its biological functions

The *KIF5B* gene, first identified in 1996, belongs to the kinesin family of proteins. These proteins facilitate the movement of organelles, proteins, and other cellular components along microtubules. *KIF5B* exhibits a structural tendency to fuse with kinase genes, such as *RET* and *ALK*, leading to the formation of chimeric proteins with constitutive activation that persistently drive downstream signaling pathways, including MAPK and PI3K/AKT (14). The chromosomal region harboring *KIF5B* may contain fragile sites prone to breakage during DNA replication or repair, increasing the likelihood of rearrangements with other genes. For instance, *KIF5B-RET* fusions result from breakage and rearrangement of the short arm of chromosome 10 (14). This fusion brings together the *RET* kinase domain and the coiled-coil domain of *KIF5B*, producing a constitutively active tyrosine kinase. Similarly, *ROS1* and *MET* can fuse with the 5’ end of *KIF5B* via their kinase domains, thereby activating downstream signaling pathways. The gene structure, chromosomal location, compatibility of functional domains, transcriptional regulation, and cell type specificity of *KIF5B* collectively contribute to its frequent rearrangement with other genes, forming a fusion gene with carcinogenic potential (14–16).

*KIF5B* is particularly important in cell division, ensuring the even distribution of organelles to daughter cells during mitosis, thereby supporting normal cell proliferation and function (15, 17).

### 3.2 *KIF5B-ALK* fusion gene

In NSCLC, *KIF5B* can fuse with *ALK*, forming the *KIF5B-ALK* fusion gene. Compared with the more common *EML4-ALK* fusion, *KIF5B-ALK* is a rare genetic alteration. Prior research demonstrated that overexpression of *KIF5B-ALK* in mammalian cells could enhance proliferation, migration, and invasion (8). This fusion protein activates key oncogenic signaling pathways, including:

PI3K/Akt pathway: It promotes cell survival and anti-apoptotic mechanisms, increasing resistance to therapy.

RAS-RAF-MEK-ERK pathway: It drives cell proliferation, migration, and cell cycle regulation.

JAK/STAT pathway: It contributes to cell proliferation and immune evasion.

Additionally, *KIF5B-ALK* fusion promotes the transition from the G1 to the S phase by activating cyclin-related proteins, such as cyclin D1, further facilitating tumor progression (8, 18).

Most *ALK* fusion mutations involve breakpoints at exon 20 of *ALK*. Different fusion partners influence the sensitivity of *ALK* fusions to tyrosine kinase inhibitors (ALK-TKIs) (19). Various *KIF5B-ALK* fusion breakpoints have been reported, each exhibiting different responses to targeted therapies:

*KIF5B-ALK (K24:A19)*: Takeuchi et al. (7) identified this fusion in a lung adenocarcinoma case (**Table 1**, case S).

**Table 1:** Summary of cases with *KIF5B-ALK* gene fusion.

Patient	Gender	Age at presentation/year	Smoking status	Diagnosis	Stage	Gene fusion	Treatment	Metastasis (part involved)	Follow-up
N1	Male	74	No	Lung adenocarcinoma	IVB	KIF5B-ALK (K20:A20)	crizotinib and ceritinib	Liver, bone, and brain	PFS of 11 months ND of 9 months
N2	Male	71	Yes	Lung adenocarcinoma	IA	KIF5B-ALK (K15:A20)	Surgery	lungs and cervical lymph nodes	Died 25 months after undergoing surgery
N3	Male	73	Yes	LCNEC	IVB	KIF5B-ALK (K17:A20)	crizotinib and alectinib	abdominal, pelvic, and brain	SD
N4	Female	18	NS	ALK-positive histiocytosis.	–	KIF5B-ALK (K24:A20)	local resection and PET	umbilicus	ND 18 months
N5	Male	51	No	ALK-positive histiocytosis.	–	KIF5B-ALK	Alectinib	Lung, brain, Multiple lymph nodes	PR 10months
S	–	–	–	Lung adenocarcinoma	–	KIF5B-ALK (K24:A19)	–	–	–

1.ND, no disease; 2.NS, not state; 3.LCNEC, large cell neuroendocrine carcinoma 4.SD, stable disease 5.PR, partial remission 6.PET, positron emission tomography; 7.S, specimens from patient with lung adenocarcinoma.

*KIF5B-ALK (K15:A20)*: Wong et al. (8) first reported this fusion in a 71-year-old female with a 5 pack-year smoking history who had quit smoking 30 years prior. The patient was diagnosed with primary lung adenocarcinoma with a maximum tumor diameter of 3 cm. Fourteen months after surgery, recurrence was found in the lung and neck lymph nodes. Without further treatment, the patient succumbed 25 months post-surgery (**Table 1**, case N2).

*KIF5B-ALK (K20:A20)*: Zeng et al. (20) described a lung adenocarcinoma patient with this fusion who responded to crizotinib, achieving 11 months of PFS before intracranial progression. Upon detection of an *ALK L1196M* mutation, ceritinib was administered, maintaining efficacy for an additional 9 months. Intracranial lesions showed partial remission (PR) without significant drug-related adverse effects (**Table 1**, case N1).

*KIF5B-ALK (K17:A20)*: This fusion was first identified in a large-cell neuroendocrine tumor. The patient developed nausea after 8 months of continuous crizotinib use and later experienced gait disturbances 10 months after drug withdrawal. Disease stability was maintained for 4 months following alectinib treatment (21) (**Table 1**, case N3). Consequently, lung cancer patients with different *KIF5B-ALK* fusion mutations have demonstrated varying degrees of response to first-generation crizotinib and second-generation TKIs, such as ceritinib and alectinib.

### 3.3 The efficacy of lorlatinib

Loratinib was approved for second-line and subsequent treatment of ALK-positive metastatic NSCLC, and its indication has since expanded to include first-line treatment (22). It has demonstrated remarkable efficacy in patients with baseline brain metastases, achieving durable intracranial responses. However, current evidence on lorlatinib’s effectiveness in patients with non-EML4 ALK fusion variants remains limited and is primarily focused on ROS1-positive NSCLC. In one reported case, a patient with advanced lung adenocarcinoma harboring ROS1 rearrangement achieved disease remission following second-line lorlatinib treatment (23). Nonetheless, clinical experience has shown that prolonged lorlatinib use may induce complex ALK resistance mutations, such as G1202R or I1171N/S/T. Ongoing research concentrates on developing more selective lorlatinib analogues through structural modifications to overcome acquired resistance (24).

In this case, the patient’s immunohistochemical analysis confirmed a primary invasive lung adenocarcinoma with high proliferative activity (Ki-67, 80%), indicating aggressive biological behavior. Studies have demonstrated that patients with concurrent *ALK* mutations experience significantly shorter PFS and lower ORRs after immunotherapy compared with those with wild-type tumors (25). Cai et al. (26) reported that although EML4-ALK mutations are associated with high PD-L1 expression, they do not correlate with increased infiltration of effector T cells, which are critical for anti-tumor immune responses. Numerous clinical studies have confirmed that immunotherapy is largely ineffective in NSCLC patients with EML4-ALK mutations and may even increase the risk of developing resistance to subsequent targeted therapies. Modulating the tumor immune microenvironment may help enhance the sensitivity of these patients to later TKI treatments (27). The intracranial and systemic lesions achieved PR after four months of postoperative lorlatinib treatment and sustained SD after eight months of continuous lorlatinib therapy. The primary AE was grade 1 limb edema. Most lorlatinib-related side effects were grade 1 or 2, while the most common grade 3 AEs were hypercholesterolemia and hypertriglyceridemia. The dose reduction rate due to AEs was 23%, the temporary withdrawal rate was 62%, and the permanent withdrawal rate was 11%. Notably, dose reductions during the first 16 weeks did not compromise lorlatinib’s efficacy (12, 28). In a phase 1/2 study (29), lorlatinib demonstrated cerebrospinal fluid (CSF) to free plasma concentration ratios ranging from 0.61 to 0.96, significantly higher than those observed with alectinib (0.002–0.005) and crizotinib (0.0006–0.026), indicating its efficient penetration across the blood-brain barrier. The remarkable intracranial response in this patient provides further clinical evidence, supporting the efficacy of lorlatinib in ALK-positive NSCLC patients with central nervous system involvement. A five-year analysis from the phase 3 CROWN study, presented at the 2024 American Society of Clinical Oncology (ASCO) meeting, reported that the median PFS for lorlatinib had not yet been reached. This prolonged PFS highlights the drug’s strong systemic and intracranial efficacy, significantly improving outcomes for patients with ALK-positive advanced NSCLC. Among patients with *TP53* mutations, lorlatinib achieved a median PFS of 51.6 months versus 5.7 months with crizotinib. In patients without *TP53* mutations, the median PFS for lorlatinib-treated cases remains unreached (12). *TP53* is a tumor suppressor gene, and its mutation or deletion can result in genomic instability and uncontrolled cell proliferation. The presence of *TP53* mutations is generally associated with a poor prognosis and reduced efficacy of TKI therapy in ALK-positive NSCLC patients (23). In the present case, the patient harbored a *TP53* frameshift mutation, which could introduce premature stop codons, leading to truncated and potentially non-functional protein products. The patient achieved SD after 8 months of lorlatinib treatment. Ongoing follow-up is required to further assess the patient’s tumor response and disease progression.

### 3.4 Limitations

This report provided an overview of several *KIF5B-ALK* fusion mutations with different breakpoints, including a case of advanced lung adenocarcinoma with *KIF5B-ALK* (K15:A20) fusion mutation that responded favorably to lorlatinib. However, several limitations are noteworthy. Firstly, data on the response of other *KIF5B-ALK* breakpoint variants to lorlatinib are lacking, and the mechanisms underlying differential sensitivity to ALK-TKIs remain elusive. Additionally, the presence of multiple genetic mutations in this patient was not considered when evaluating treatment efficacy. The impact of coexisting *TP53* mutation on lorlatinib’s effectiveness warrants further investigation. Long-term follow-up is essential to assess overall survival and quality of life in patients with this fusion mutation.

## 4 Conclusion

This report described a rare case of advanced lung adenocarcinoma with an *ALK* fusion and a high number of genetic mutations. The patient, harboring 52 genetic variants, including the *KIF5B-ALK* (K15:A20) fusion, achieved prolonged disease control with lorlatinib following intracranial surgery. This case provided valuable clinical evidence, supporting the use of lorlatinib in patients with the *KIF5B-ALK* (K15:A20) fusion mutation.

## Data Availability

The original contributions presented in the study are included in the article/supplementary material. Further inquiries can be directed to the corresponding author.
